# Supplementary material for: Signaling Through FcγRIIA and the C5a-C5aR Pathway Mediate Platelet Hyperactivation in COVID-19
Source: Front Immunol. 2022 Mar 3;13:834988. doi: 10.3389/fimmu.2022.834988 (PMC8928747; doi:10.3389/fimmu.2022.834988)
Supplement: Supplementary file 1 [file DataSheet_1.docx]

**Supplementary materials:**

**
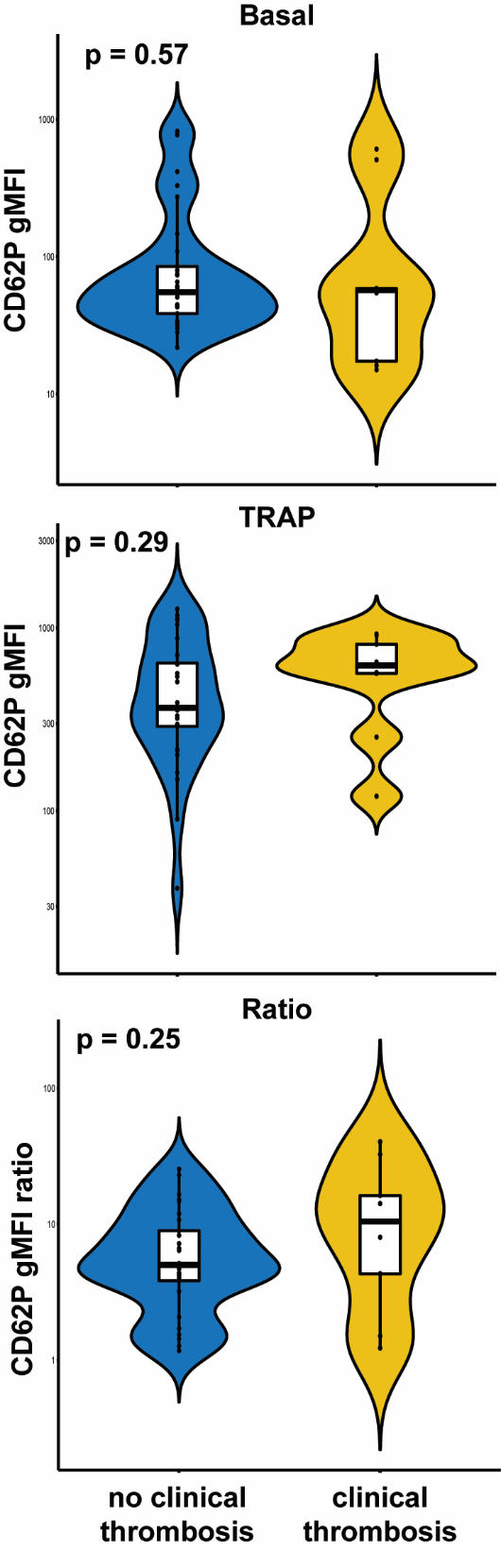
Supplementary Figure 1.**

**Supplementary Figure 1.** Cumulative data for CD62P surface expression of *ex vivo* isolated platelets at baseline (basal), after TRAP activation (TRAP) and their ratio for hospitalized COVID-19 patients that experienced a clinical thrombosis or not. Mann-Whitney non-parametric testing was used and the *p*-values are depicted.

**Supplementary Figure 2.**

**
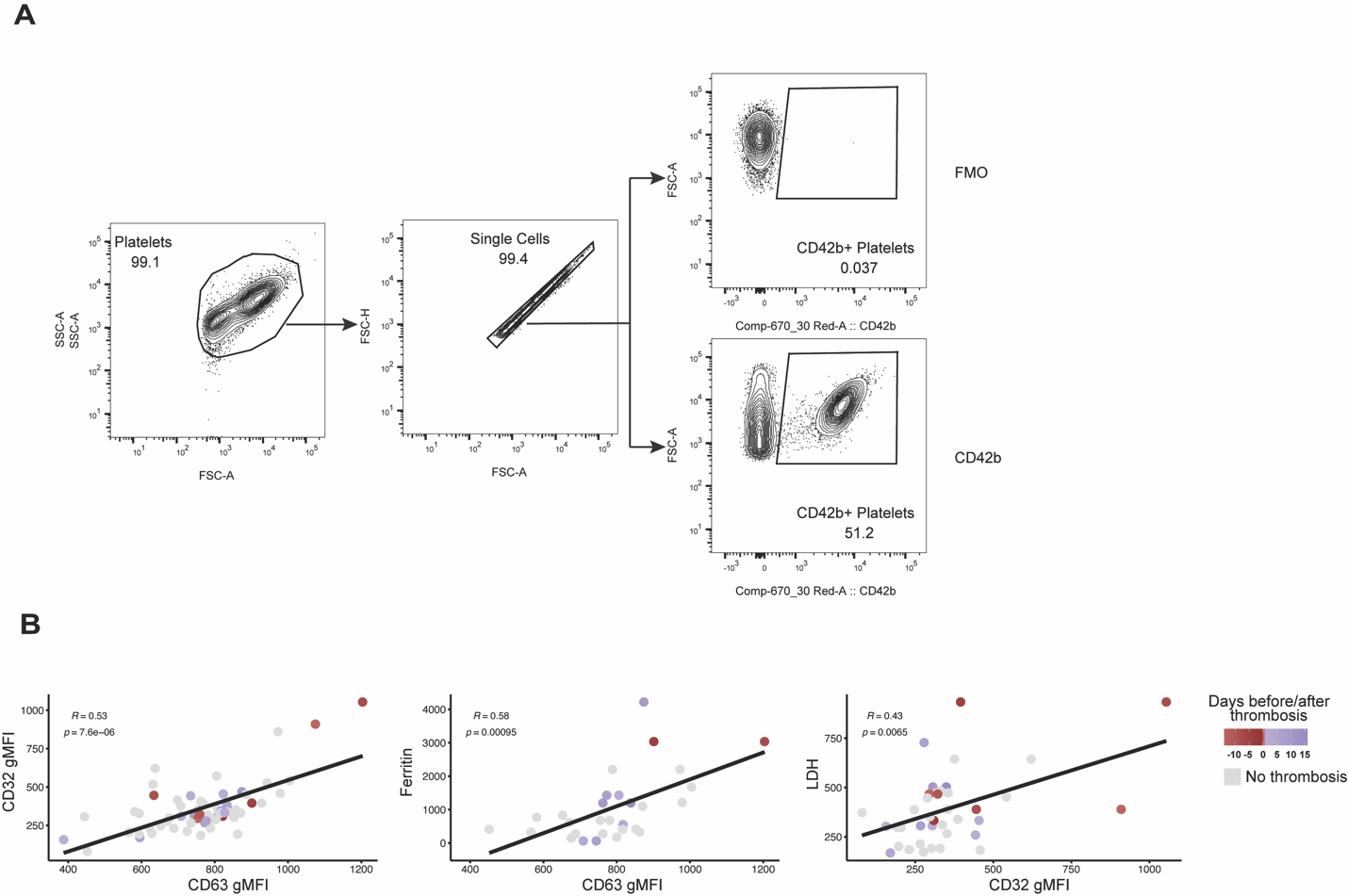
**

**Supplementary Figure 2. A)** Gating strategy for isolated control platelets incubated with COVID-19 plasma. **B)** Representative scatter plots for CD32 vs CD63, ferritin vs CD63 and LDH vs CD32. Samples are colored based on the absence (grey color) or presence of thrombosis relative to the time of draw (red before thrombosis and blue after thrombosis). The Spearman correlation co-efficient and the corresponding *p*-value are depicted.

**Supplementary Figure 3.**


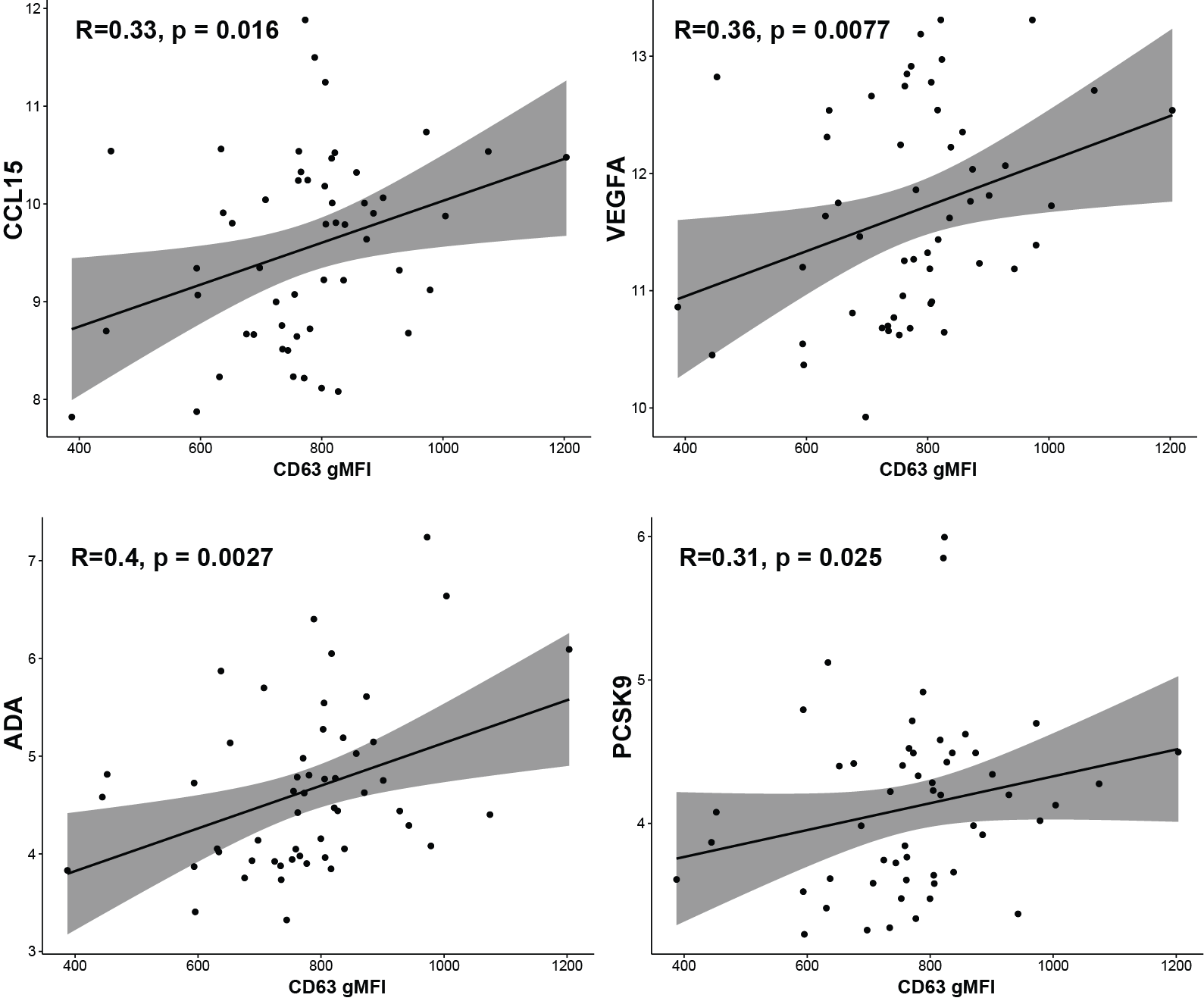


**Supplementary Figure 3.** Correlation plots for CD63 gMFI with representative analytes CCL15, VEGFA, ADA and PCSK9 from Figure 3 (n=53).

**Supplementary Figure 4.**

**
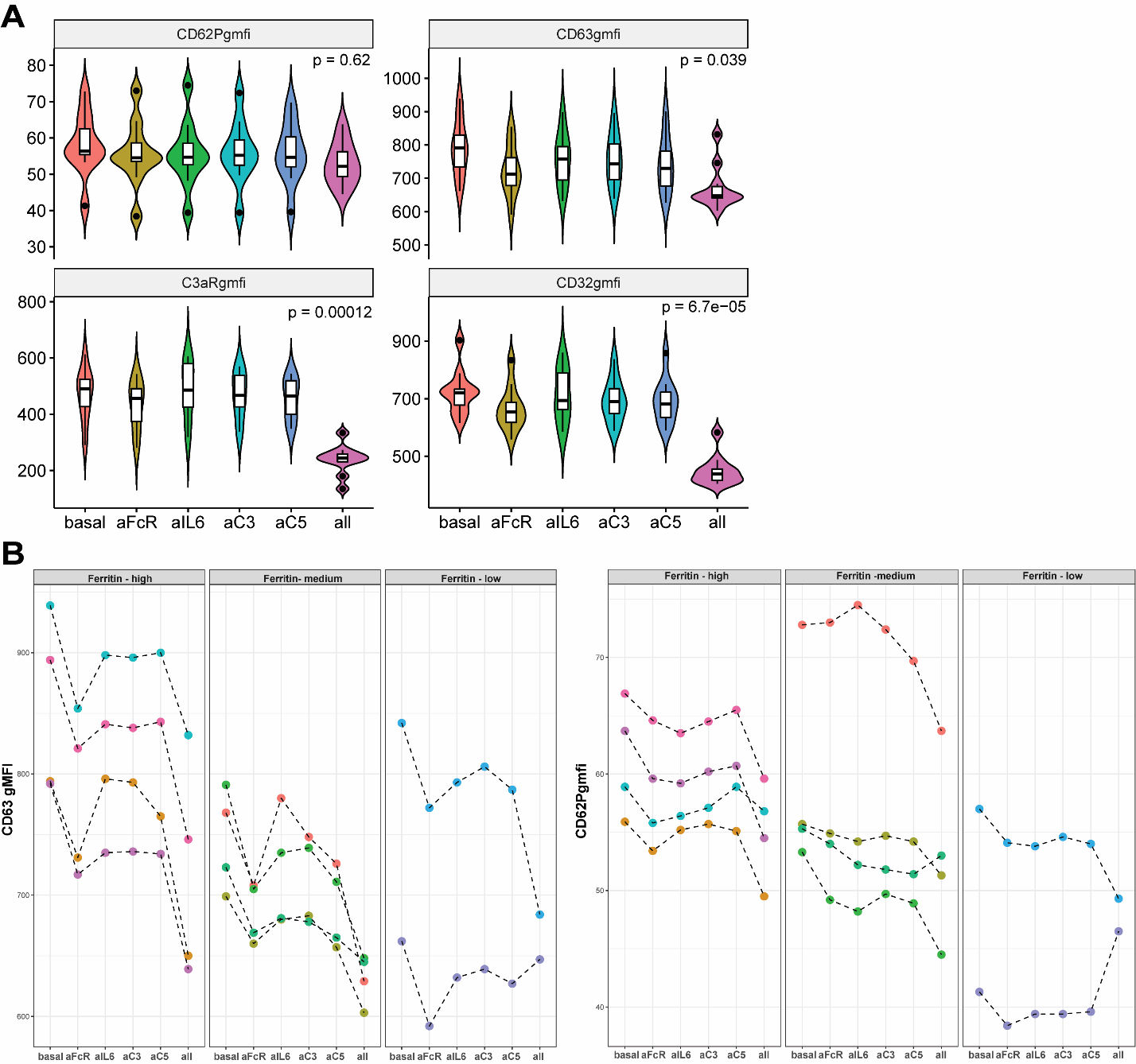
**

**Supplementary Figure 4. A)** Violin plots of gMFI expression of CD62P, CD63, CD32 and C3aR on the surface of control platelets incubated with COVID-19 plasma (n=10 patients) in the presence or absence of neutralizing antibodies to FcγRIIa, IL6, C3a and C5a, as indicated. Kruskal-Wallis non-parametric testing was used to compare the groups and the *p*-values are depicted. **B)** Same samples and conditions depicted in (A) but categorized based on the corresponding patient’s ferritin levels. Ferritin – low: <1000ng/mL; Ferritin – medium: 1000-2000ng/mL; Ferritin – high >2000ng/mL

**Supplementary Figure 5.**

**
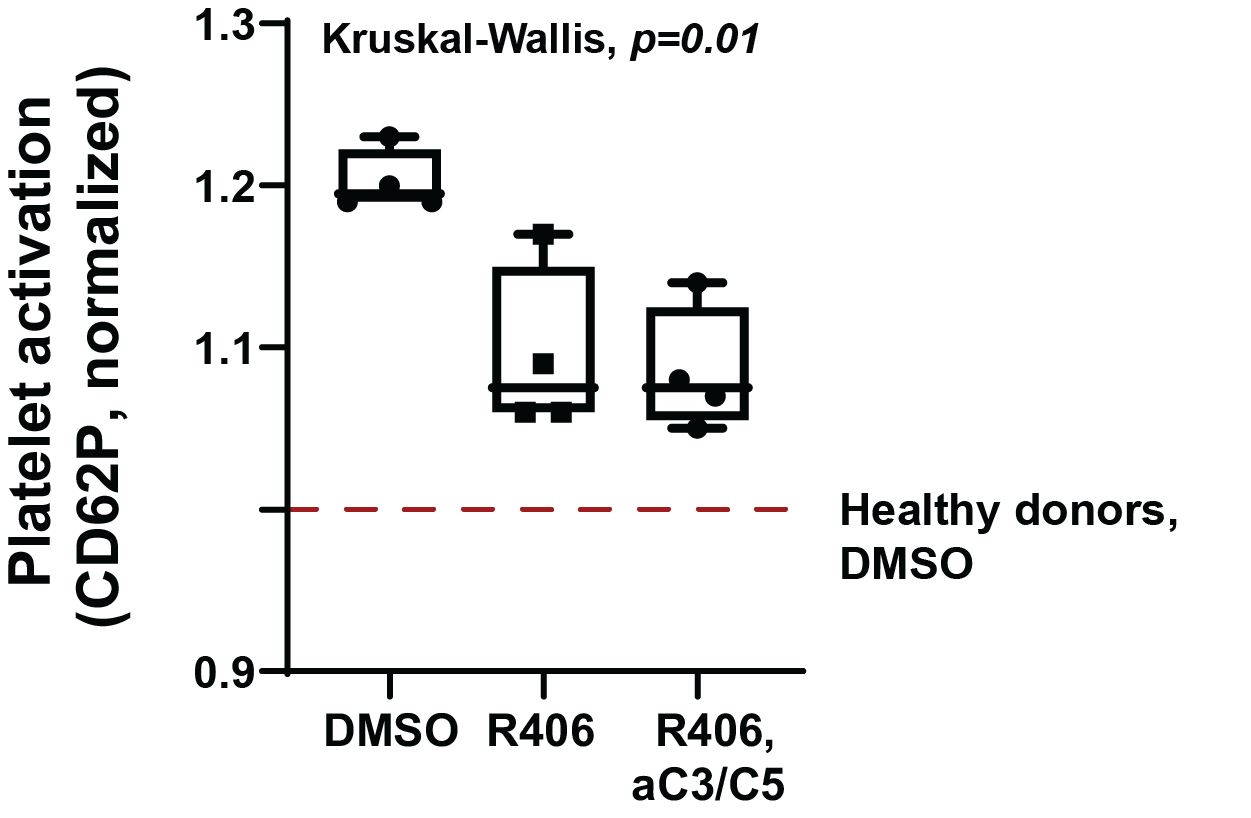
**

**Supplementary Figure 5.** Boxplots of gMFI expression of CD62P on the surface of healthy control platelets incubated with COVID-19 plasma (n=4 patients) in the absence or presence of fostamatinib or fostamatinib and neutralizing antibodies to C3a and C5a. Kruskal-Wallis non-parametric testing was used to compare the groups and the *p*-value is depicted.

**Supplementary Table 1.**

|  | COVID-19 patients with platelets  assayed *ex vivo*  (33 unique patients, 38 samples) | COVID-19 patients with plasma  assayed *in vitro*  (47 unique patients, 63 samples) |
| --- | --- | --- |
| Age (mean, SEM) | 60.2 (2.94) | 58.7 (2.14) |
| Gender (male, %) | 48% | 62% |
| BMI (mean, SEM) | 34 (2.2) | 32.7 (1.67) |
| Platelet count (mean, SEM) | 261.9 (17.8) | 246 (12.6) |
| WBC count (mean, SEM) | 7.9 (0.6) | 9.3 (1.0) |
| D-dimer (mean, SEM) | 4.37 (1.64) | 7.04 (2.8) |
| Ferritin (mean, SEM) | 1350.3 (363) | 1650.1 (313.8) |
| LDH (mean, SEM) | 347.2 (22.9) | 404.5 (33.25) |
| Hs-CRP (mean, SEM) | 91.8 (10.9) | 81.53 (12.57) |
| CVD risk (%) | 82% | 87% |
| APACHEIII (mean, SEM) | 61.6 (3.9) | 63.58 (4.27) |
| Enrollment NIH Disease Severity Score (mean, SEM) | 3.4 (0.18) | 3.28 (0.15) |
| Incident thrombosis (%) | 9% | 34% |
| SARS-CoV-2 IgM (ug/mL, mean, SEM) | 10.47 (3.73) | 10.53 (4.48) |
| SARS-CoV-2 IgG (ug/mL, mean, SEM) | 41.1 (8.76) | 47.9 (10.65) |

**Supplementary Table 1.** Clinical information of the COVID-19 patients evaluated.

**Supplementary Table 2**.

|  | Condition | Fold-change  (mean ± SEM) | P value vs.  No drug |
| --- | --- | --- | --- |
| **Immediate** | --- | 55.85 ± 29.63 |  |
|  | R406 | 2.61 ± 0.92 | 0.0255 |
|  | Anti-FcR | 0.96 ± 0.34 | 0.0245 |
|  | Anti-C5a | 1.35 ± 0.50 | 0.0275 |
| **5 mins** | --- | 406.65 ± 363.08 |  |
|  | R406 | 5.613 ± 2.68 | 0.0255 |
|  | Anti-FcR | 3.08 ± 2.36 | 0.0245 |
|  | Anti-C5a | 9.79 ± 9.14 | 0.0275 |
| **10 mins** | --- | 762.60 ± 719.81 |  |
|  | R406 | 6.68± 3.373 | 0.0255 |
|  | Anti-FcR | 4.13 ± 3.34 | 0.0245 |
|  | Anti-C5a | 17.52 ± 16.98 | 0.0275 |
| **15 mins** | --- | 415.88 ± 371.80 |  |
|  | R406 | 5.86 ± 2.92 | 0.0255 |
|  | Anti-FcR | 3.75 ± 1.88 | 0.0245 |
|  | Anti-C5a | 9.13 ± 8.54 | 0.0275 |

**Supplementary Table 2**. Inhibition of platelet aggregation in hematoporphyrin-induced photochemical injury model in an endothelial-lined microfluidic channel. Analysis of relative fluorescence intensity of platelet aggregation in the hematoporphyrin-induced photochemical injured endothelial-lined microfluidic channel at different times after infusion of platelets in plasma (n=7) from a severe COVID-19 patient: immediate, 5 min, 10 min and 15 min. Data is expressed as fold increase of platelet accumulation with patient plasma over healthy donor plasma. P values were calculated using Dunnett’s multiple comparisons test, n=7.
